# A Lower Level of Post-Vaccinal Antibody Titer against Influenza Virus A H1N1 May Protect Patients with Autoimmune Rheumatic Diseases from Respiratory Viral Infections

**DOI:** 10.3390/medicina58010076

**Published:** 2022-01-04

**Authors:** Milomir S. Milanovic, Djordje M. Kadijevich, Ljudmila Stojanovich, Branislav Milovanovic, Aleksandra Djokovic

**Affiliations:** 1 Clinic for Infectious and Tropical Diseases, Military Medical Academy, Crnotravska 17, 11040 Belgrade, Serbia; 2 Institute for Educational Research, Dobrinjska 11/III, 11000 Belgrade, Serbia; djkadij@mi.sanu.ac.rs; 3 Department of Internal Medicine, University Medical Centre “Bezanijska Kosa”, Dr Žorža Matea bb, 11080 Belgrade, Serbia; ljudmila_stojanovich@yahoo.com (L.S.); branislav_milovanovic@vektor.net (B.M.); drsaska@yahoo.com (A.D.)

**Keywords:** autoimmune rheumatic diseases, clinical effectiveness of influenza vaccination, personal/health-related conditions, respiratory viral infections, sero-protective titer

## Abstract

*Background and Objectives:* The concentration of antibodies against virus influenza A H1N1 in the titer (≥1:32) positively correlates with resistance to flu in healthy persons. In elderly and immune-compromised patients, an influenza vaccine may be less immunogenic. *Hypothesis*: A lower post-vaccinal antibody titer (≥1:16) may be sero-protective against respiratory viral infections in patients with autoimmune rheumatic diseases. *Materials and Methods*: Fifty patients with autoimmune rheumatic diseases (Systemic Lupus Erythematosus—24; Rheumatoid Arthritis—15; and Sjögren’s Syndrome—11), who were at least 65 years old or whose relative disease duration (disease duration/age) was greater than 1/8, were examined. Thirty-four of them were vaccinated with a trivalent inactivated non-adjuvant influenza vaccine. The antibody concentration against influenza virus A H1N1 was measured using the standardized hemagglutination inhibition test and patients who got any respiratory viral infection were registered. To test the hypothesis, a correlative analysis was applied, followed by a binary logistic regression that included potential confounding variables, such as age, disease duration and therapy (personal/health-related conditions). *Results*: Vaccinated patients were significantly less affected by respiratory viral infections (21% vs. 75%). The lower titer considered (≥1:16) was significantly present more often among vaccinated patients (68% vs. 6%). The correlation between its presence/absence and that of respiratory viral infections was –0.34 (*p* < 0.05). The binary logistic regression evidenced the relevance of this correlation, confirming the hypothesis. Vaccination was associated with the 87.3% reduction in the likelihood of getting respiratory viral infections, whereas the lower antibody titer (≥1:16) was associated with the 77.6% reduction in the likelihood of getting respiratory viral infections. The vaccine was well tolerated by all patients and after vaccination no exacerbation of the underlying disease was observed. *Conclusions*: A lower antibody titer (≥1:16) against influenza virus A H1N1 could be protective against respiratory viral infections for certain autoimmune rheumatic diseases patients, which confirms the clinical effectiveness of influenza vaccination.

## 1. Introduction

Influenza causes a wide variety of respiratory diseases in the human population, ranging from sub-clinical forms of infection to fulminant primary viral or secondary bacterial pneumonia; it is responsible for the death of about half-a-million patients worldwide every year. Despite the growing availability of numerous antiviral drugs, seasonal influenza vaccination is the most effective strategy in the fight against respiratory infections. The vaccine response is determined by different predictive factors, including the genetic or constitutional characteristics of each individual patient (https://www.who.int/news-room/fact-sheets/detail/influenza-(seasonal), accessed on 27 December 2021).

Owing to certain immuno-regulatory disorders, as well as the use of immuno-modulating drugs (corticosteroids, disease-modifying anti-rheumatic drugs (DMARDs) and biological medicines), patients with autoimmune rheumatic diseases (AIRDs) are at increased risk of flu and severe respiratory complications. Therefore, an influenza vaccination should be carefully considered and recommended at the right time—in a stable phase of the underlying disease—simultaneously bearing in mind the immunogenicity of the vaccine and/or its potentially harmful effects [1,2,3,4]. When vaccinations are administered to chronically diseased persons, it is reasonable to expect that, in measuring effectiveness against viral infections, sero-protective titers may differ from those found in healthy persons, as explained below based on several earlier studies. The level of protection is determined by the amount and avidity of neutralizing antibodies; whether these were pre-existing or induced by the vaccine is irrelevant [5].

The concentration of antibodies in the titer (≥1:40) after an influenza vaccination is often assumed protective against flu in healthy adults (e.g., [6]), meaning that this level of antibodies positively correlates with resistance to flu. The concentration of antibodies (≥1:40) represents a four-fold rise in titers from the initial 1:10 dilution used in the serological tests. When the base 1:8 dilution is applied, a four-fold rise in titers gives the concentration of ≥1:32 and may be considered as sero-protective, as conducted in other studies (e.g., [7,8]).

Research evidences that patients with autoimmune rheumatic diseases receiving immunosuppressive or biological therapy may have a decreased level of humoral response after flu vaccination [9,10,11,12]. Such a level of antibodies might be the result of an abnormal basic immune system response—the immunosuppressive effect of the underlying AIRD and the occurrence of a “locus minoris resistentiae” as a sequel to the AIRD [2]. Additionally, in elderly and immuno-compromised patients, an influenza vaccine may be less immunogenic than in younger healthy individuals [13,14,15,16]. To examine this deficiency, focusing on the population aged 65 or older might be suitable, as performed in several studies on vaccine immunogenicity and effectiveness, as well as health care costs (e.g., [17,18]); including relative disease duration may also be relevant (cf. [19]). Although more research is needed on the extent of immuno-suppression in relation to disease activity, different medication combinations, sex, age and disease duration [20], a post-vaccinal antibody titer against influenza virus A H1N1 might just be present at a lower level (e.g., ≥1:16) instead of at a higher one (e.g., ≥1:32), due to some personal/health-related conditions. Then, an important question is whether this lower level can be protective against infections.

Consideration of a lower level of post-vaccinal antibody titer as protective was suggested by a recent PhD study [21] involving patients who suffered from Systemic Lupus Erythematosus (SLE), Rheumatoid Arthritis (RA) and Sjögren’s Syndrome (SS). The correlation between the presence/absence of the assumed sero-protective titer (SPT ≥ 1:32) and that of respiratory viral infection (RVI) was not as significantly negative as one could expect (the presence of SPT could be coupled with the absence of these infections, statistically) but non-significant and almost zero. This finding initiated additional research, whose outcome is presented in this paper.

### Hypothesis

A post-vaccine immune response could be influenced by some personal/health-related conditions, such as age, disease duration and therapy. Thus, we postulated the following hypothesis: A lower level of antibody titer against influenza virus A H1N1 (≥1:16) may be protective against RVIs for AIRD patients, provided that some personal/health-related conditions hold true.

**Table 1:** Descriptive and inferential statistics by variable.

Variable		Descriptive Statistics ^1^				Inferential Statistics
Type	Name	All	SLE	RA	SS	Comparing SLE, RA and SS
Continuous	Age	60.4 (11.2) ^2^	54.5 (10.5)	65.6 (7.9)	66.1 (10.5)	F(2,47)=8.394,p=0.001
	Disease duration	11.6 (5.8)	13.0 (3.2)	13.1 (7.7)	6.5 (4.7)	F(2,47)=6.779,p=0.003
	Relative disease duration	0.20 (0.10)	0.24 (0.07)	0.20 (0.12)	0.11 (0.09)	F(2,47)=8.377,p=0.001
Binary	Vaccination 5	30%	25%	46.7%	18.2%	$\chi^{2}=3.001,df=2,p=0.223$
	Vaccination 4	30%	25%	46.7%	18.2%	$\chi^{2}=3.001,df=2,p=0.223$
	Vaccination 3	36%	33.3%	46.7%	27.3%	$\chi^{2}=1.178,df=2,p=0.555$
	Vaccination 2	44%	50%	46.7%	27.3%	$\chi^{2}=1.643,df=2,p=0.440$
	Vaccination 1	56%	54.2%	73.3%	36.4%	$\chi^{2}=3.583,df=2,p=0.167$
	**Vaccination** **(main)**	**68%**	**66.7%**	**73.3%**	**63.6%**	$\chi^{2}=0.312,\mathrm{df}=2,p=0.856$
	Gender	82%	83.3%	66.7%	100%	$\chi^{2}=4.833,df=2,p=0.089$
	Smoking	22%	20.8%	26.7%	18.2%	$\chi^{2}=0.303,df=2,p=0.859$
	Bronchitis	20%	20.8%	6.7%	36.4%	$\chi^{2}=3.518,df=2,p=0.172$
	Therapy	70%	79.2%	80%	36.4%	$\chi^{2}=7.601,df=2,p=0.022$
	**Respiratory** **viral** **infection**	**38%**	**41.7%**	**26.7%**	**45.5%**	$\chi^{2}=1.214,\mathrm{df}=2,p=0.545$
Categorical	Titer	8 ^3^	12	0	8	$\chi^{2}=0.078,df=2,p=0.962$
Binary	Assumed SPT	36%	41.7%	26.7%	36.4%	$\chi^{2}=0.902,df=2,p=0.637$
	**Personal** **SPT**	**48%**	**50%**	**46.7%**	**45.5%**	$\chi^{2}=0.078,\mathrm{df}=2,p=0.962$

^1^ Concerning all patients as well as those with specific disease. ^2^ M = 60.4 years (SD = 11.2 years). ^3^ Median.

## 2. Materials and Method

### 2.1. Sample

We used a cross-sectional study with a convenience sample of 50 AIRD patients (SLE—24; RA—15; and SS—11), which was of an appropriate size for the expected correlation of 0.4 under α = 0.05 and β = 0.2 (see http://www.sample-size.net/correlation-sample-size/, accessed on 27 December 2021, for a sample size calculator). These patients were selected from the 66-patient rheumatic reference database used in the PhD study mentioned above [21], which, as already mentioned, included SLE, RA and SS patients. The present study only considered patients who were at least 65 years old or whose relative disease duration (with respect to age) was greater than 1/8 (0.125); relative disease duration for a patient aged 60, who had been diseased for 15 years, was 15/60 = 0.25. To obtain a sample of an appropriate size, the cut value of 0.125 was applied, which was close to the 40th percentile for all patients in the database. These fifty participants were considered as one group, because, as evidenced by Table 1 on the previous page, patients with SLE, RA and SS did not differ with respect to most variables examined in this study, especially those variables indicated in bold.

### 2.2. Design and Variables

This study primarily used a correlative design. The variables were:

- Age (in years);
- Disease duration (in years);
- Relative disease duration (defined by disease duration/age);
- Gender (1—female; 0—male);
- Smoking (1—smoker; 0—non-smoker);
- Therapy (1—Methotrexate and/or glucocorticoids; 0—DMARDs and/or glucocorticoids);
- Vaccination (1—vaccinated; 0—non-vaccinated);
- Vaccination 1 (1—vaccinated a year ago; 0—otherwise);
- Vaccination 2 (1—vaccinated two years ago; 0—otherwise);
- Vaccination 3 (1—vaccinated three years ago; 0—otherwise);
- Vaccination 4 (1—vaccinated four years ago; 0—otherwise);
- Vaccination 5 (1—vaccinated five years ago; 0—otherwise);
- Bronchitis (1— had it before the main vaccination; 0— otherwise);
- Respiratory viral infection (RVI) (1—got it after the main vaccination; 0—otherwise);
- Titer (with values 0, 8, 16, 32, …., 1024);
- Assumed sero-protective titer (assumed SPT) (1—Titer ≥ 1:32; 0—otherwise);
- Personal SPT (1—Titer ≥ 1:16; 0—otherwise).

Many of these variables are possible vaccine efficacy factors and their values were collected in previous epidemiological studies and taken from the patients’ hospital records.

### 2.3. Treatment and Procedure

On a voluntarily basis and during a stable status of their diseases, thirty-four patients were vaccinated with a trivalent inactivated non-adjuvant influenza vaccine (15 μg of HA A/California/7/2009 (H1N1), 15 μg of HA A/Pert/16/2009 (H3N2) and 15 μg of HA B/Brisbane/60/2008). Thus, the control group consisted of sixteen patients who did not accept the proposed vaccination.

The antibody concentration (titer) against influenza virus A H1N1 was measured two months later in both vaccinated and non-vaccinated patients by the standardized method (HIA)—a hemagglutination inhibition assay was used at a dilution of 1:8 to 1:1024 (according to the CDC method, with A/California/7/2009 (H1N1) flu virus antigen and turkey erythrocytes). We considered an antibody titer with a dilution of ≥1:32 to be the protective titer, originating from immunization, symptomatic or asymptomatic infection. Apart from the HIA method, we performed other serological testing (the complement fixation test or enzyme-linked immuno-sorbent essay) to identify common respiratory tract viruses (influenza virus A and B, respiratory syncytial virus, rhinovirus) and polymerase chain reaction (PCR) assay for the detection of influenza viruses. All testing was performed at “Torlak” Institute of Virology, Vaccines and Sera, which is a national, WHO (World Health Organization) reference laboratory.

During the six months following vaccinations, we examined and registered patients who had any respiratory viral (flu, sub-clinical flu, or flu-like illness) or secondary bacterial infections and potentially harmful effects of vaccination. Then, the presence of viral infections (primarily influenza in flu season) in correlation to humoral response against influenza virus A H1N1, as well as parameters of disease activity (SLE—SLEDAI; RA—DAS28; and Sjögren’s Syndrome—ESSDAI), was monitored in vaccinated and non-vaccinated patients.

### 2.4. Statistical Analysis

To test the hypothesis, a correlative analysis was applied to the two binary variables in question. The Pearson correlation coefficient was determined (for binary variables, this coefficient is equal to that of Kendall or Spearman) and its statistical difference from zero was examined using the *t*-test. This correlative analysis was followed by binary logistic regression, which, apart from these two binary variables, included potential confounding variables that might influence their relationship.

To examine possible differences among the three groups of patients in terms of the variables considered, this study used three statistical tests. Pearson chi-squared test was applied for the comparison of proportions on a contingency table. The *F*-test was used for the one-way ANOVA hypothesis, testing whether the three population means were equal. For testing whether three population medians were equal (Mood’s median test–a nonparametric alternative to the one-way ANOVA), a Pearson chi-squared test was applied.

## 3. Results

The trivalent inactivated non-adjuvant influenza vaccine was well tolerated by all SLE, RA and SS patients and, after the main vaccination, no exacerbation of the underlying disease was observed.

According to Table 1, SLE, RA and SS patients did not differ with respect to most variables examined in this study. Statistically significant differences, underlined in the table, were found for age, disease duration and relative disease duration. Such differences were also found for therapy (*p* < 0.05) and gender (*p* < 0.1), mostly resulting from the specificities of our SS patients (all female patients mostly treated with DMARDs and/or glucocorticoids), as well as the fact that SLE and RA patients were mostly treated with Methotrexate and/or glucocorticoids.

**Figure 1:**
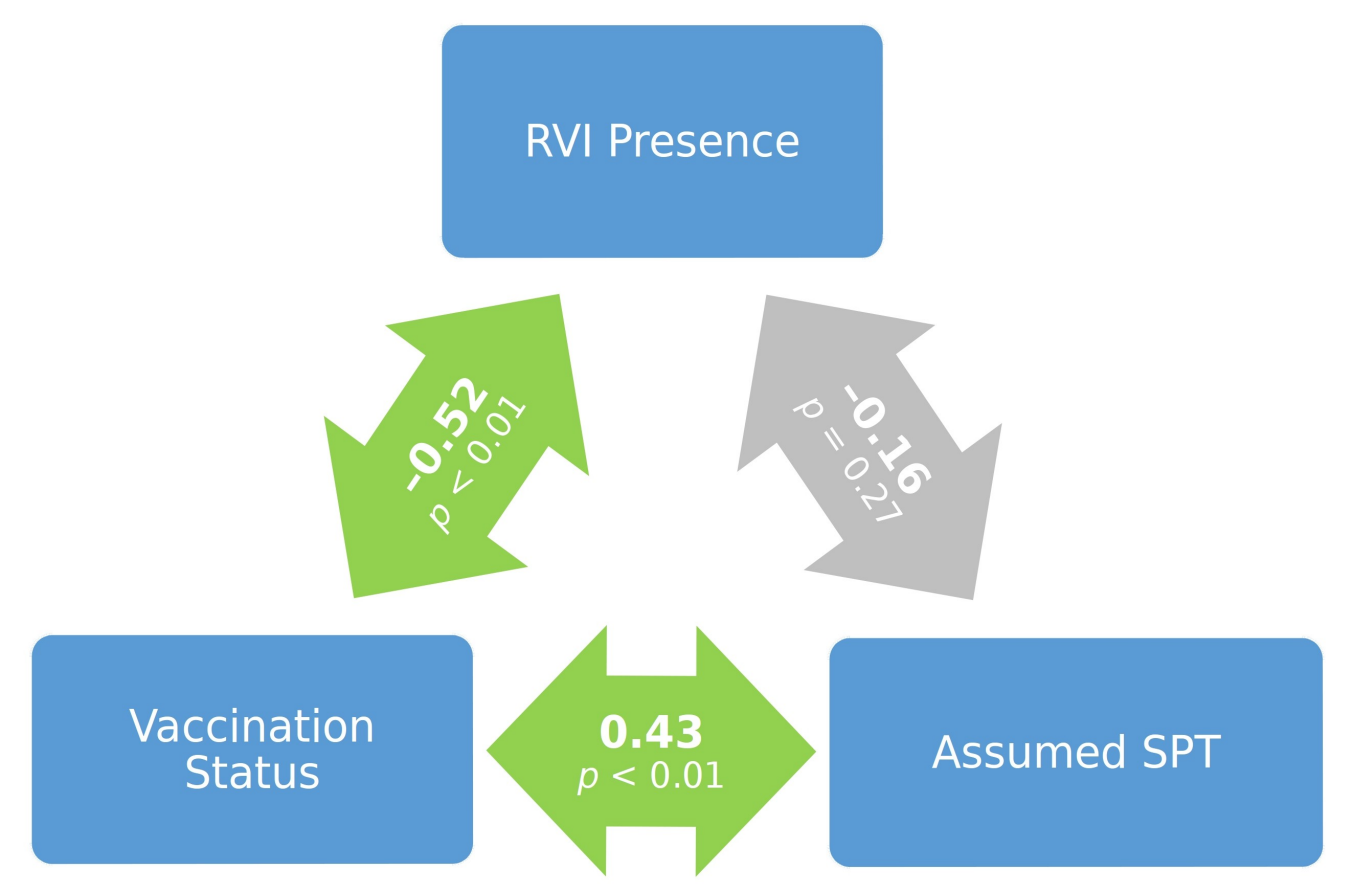
Correlations among vaccination status, assumed sero-protective titer and respiratory viral infection presence.

The vaccination efficacy was examined via the commonly assumed SPT (≥1:32) and the correlation between variables assumed SPT and RVI was non-significant and almost zero (see Figure 1). On the other hand, as expected, vaccinated patients were significantly less affected by RVIs (primarily influenza)—21% (7/34) vs. 75% (12/16) in the non-vaccinated group ($\chi^{2}$ = 13.672, df = 1, *p* = 0.000). Further, assumed SPT was significantly more often present among vaccinated patients (50% vs. 6%; $\chi^{2}$ = 9.039, df = 1, *p* = 0.003). This was a result of the following distributions of titer values for vaccinated as opposed to nonvaccinated patients: 0 (8− in 7 vs. 13); 8 (8+ in 4 vs. 2); 16 (16+ in 6 vs. 0); 32 (32+ in 3 vs. 0); 64 (64+ in 8 vs. 1); 256 (256+ in 4 vs. 0); 512 (512+ in 1 vs. 0); 1024 (1024+ in 1 vs. 0).

**Figure 2:**
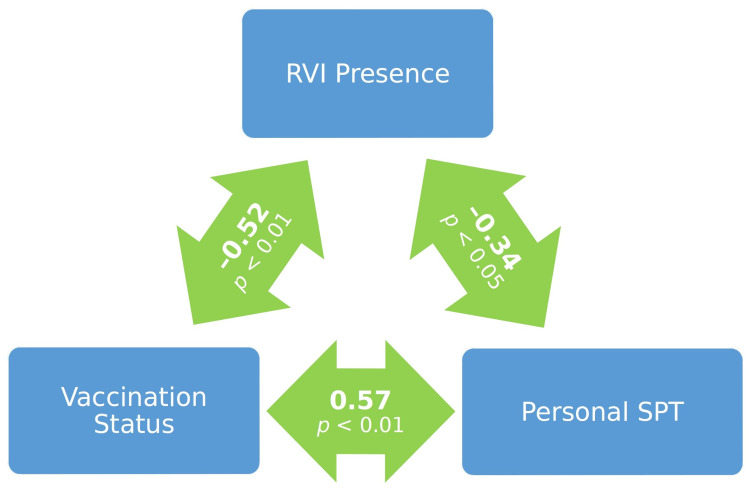
Correlations among vaccination status, personal sero-protective titer and respiratory viral infection presence.

To test the hypothesis, we simply had to examine the correlation between the variables RVI and personal SPT being used for certain AIRD patients (in particular for those who were at least 65 years old or whose relative disease duration was greater than 1/8). This correlation was negative and significant (see Figure 2). Additionally, as expected, personal SPT was significantly more often present among vaccinated patients (68% vs. 6%; $\chi^{2}$ = 16.432, df = 1, *p* < 0.001). Despite some differences among the SLE, RA and SS patients reported above, significant differences for personal SPT, such as assumed SPT, were not found (see Table 1).

Then, a forward (Wald) binary logistic regression was performed to examine the effects of personal SPT, age, disease duration, relative disease duration, gender, smoking, bronchitis, therapy, five prior vaccinations and disease (1—SLE; 2—RA; 3—SS; three dummy variables were used) on the likelihood that patients got RVIs after the main vaccination. The binary logistic regression with two predictors (personal SPT and bronchitis) was statistically significant, with $\chi^{2}$ = 19.206, df = 2, *p* < 0.001. This model explained 43.4% (Nagelkerke $R^{2}$) of the variance in RVIs, classifying 78% of cases correctly. Personal SPT was associated with the 77.6% reduction in the likelihood of getting RVI, whereas bronchitis increased that likelihood by 27.2 times.

When personal SPT was replaced by vaccination (main), the model with two predictors (vaccination and bronchitis) was statistically significant, with $\chi^{2}$ = 22.094, df = 2, *p* < 0.001. This model explained 48.6% (Nagelkerke $R^{2}$) of the variance in RVIs, classifying 80% of cases correctly. While vaccination was associated with the 87.3% reduction in the likelihood of getting RVI, bronchitis increased that likelihood by 17.4 times.

## 4. Discussion

Three important findings emerged from this study. Firstly, vaccinated patients were significantly less affected by RVIs (primarily influenza), 21% vs. 75% in the non-vaccinated group. Secondly, the sero-protective titer (≥1:16) against influenza virus A H1N1 was significantly more often present among vaccinated patients (68% vs. 6%). Thirdly, the correlation between the presence/absence of the lower antibody titer (≥1:16) and that of RVIs in our AIRD patients was –0.34 (*p* < 0.05).

Clinical multidimensional studies dealing with influenza vaccination are relatively rare. Most studies generally evaluate the immune response to influenza vaccination (i.e., the development of antibody titers) [6]. Regarding vaccinated AIRD patients, our research study has evidenced clinical effectiveness, vaccination safety and efficacy—i.e., satisfactory humoral response, as found in other studies [7,22,23,24,25,26].

Less frequent viral respiratory and secondary bacterial infections were documented in SLE and RA vaccinated patients. Vaccinated patients had significantly fewer occurrences of infections. Every viral and bacterial infection resulted in the worsening of the main disease [27]. Influenza vaccination was associated with a reduced risk of hospitalization due to septicemia, bacteremia, or viremia in SLE patients [28]. RA patients receiving the influenza vaccine have significantly lower morbidity and mortality, particularly in elderly patients [29]. In our study, the occurrence of pneumonia that required hospitalization predominantly occurred in unvaccinated patients (three unvaccinated and one vaccinated) and vaccinated patients were significantly less affected by respiratory viral infections.

One multi-centric study reports that, compared to the control patients, the prevalence of pneumonia in the flu season that required hospitalization was 2–3 times higher in RA patients and tended to be higher in regions with low vaccination rates. A lower pneumonia prevalence in East Germany indicates that influenza vaccination may help to reduce respiratory complications in RA patients [30]. Another study estimated that the influenza vaccination rate among patients with inflammatory rheumatic diseases was moderate (25%) despite current guidelines recommending this vaccination for this group of patients [31]. A study in Denmark found that, among RA patients receiving immunosuppressive drugs, there was a low prevalence of influenza vaccination [32].

The vaccination rate among the patients in our study was relatively high (68%). The vaccinated patients were significantly less affected by RVIs (primarily influenza). Vaccination efficiency was first examined via the commonly assumed SPT (≥1:32) and the correlation between the presence/absence of this SPT and that of RVI was non-significant and almost zero. When a lower concentration of antibodies against influenza virus A H1N1 (antibody titer ≥ 1:16) was examined, it was found that the correlation between the presence/absence of such a personalized SPT (personal SPT) and that of RVIs was negative and significant, provided that patients were at least 65 years old or their relative disease duration was greater than 1/8 (0.125). (Both assumed SPT and personal SPT were significantly more often present among the vaccinated patients and SLE, RA and SS patients did not significantly differ with respect to the frequency of their presence.) The applied binary logistic regression evidenced the relevance of this correlation. These results confirm the hypothesis that, for AIRD patients, a lower concentration of antibodies against influenza virus A H1N1 (antibody titer ≥ 1:16) may be protective against RVIs, provided that some personal/health-related conditions hold true. Of course, this does not exclude the protective nature of antibody titer ≥ 1:32, but this is not statistically demonstrated at a significant level in our study.

To the authors’ knowledge, a personalization of SPT has not been studied so far, neither have research studies considered the relationship (in terms of correlation) between humoral response to influenza vaccination and RVIs in AIRD patients, as conducted in this study. It may also be viewed as a valuable contribution to statistics education in medical research, because, when an expected relationship is missing, this relationship might still hold true for specific patients or particular threshold values, provided that such grouping, defendable in medical terms, could be uncovered in additional analysis (cf. [33]).

Although more research is needed on the vaccination in AIRD patients, especially concerning vaccine-preventable infectious diseases and the safety of vaccination, the use of live vaccines is generally contraindicated in patients being treated with an immunosuppressive/immunomodulatory drug because of the risk of infection that might be generated by the vaccine strains [2]. On the other hand, chronic pulmonary and other chronic diseases, as well as pulmonary complications in AIRD patients, could be important reasons for possible influenza vaccination. According to the recommendations from EULAR (European Alliance of Associations for Rheumatology), influenza and pneumococcal vaccination should be strongly considered for the majority of adult patients with AIRD [34]. In other studies within the literature review, authors highlight the importance of the efficacy and safety of influenza vaccination in patients with AIRD, especially now, during the pandemic caused by coronavirus disease 2019 (COVID-19) [35].

## 5. Conclusions

This study evidenced that a lower concentration of antibodies against influenza virus A H1N1 (antibody titer ≥ 1:16) was protective against RVIs for certain AIRD patients. This finding may open a new dimension in the interpretation of the necessary level of humoral response against influenza in AIRD patients, taking into account not only absolute but also relative disease duration. Further research, directed towards individual sero-protective response in these patients, might resolve many dilemmas and provoking questions regarding vaccination, such as its tolerance, immunogenicity and clinical effectiveness, contributing to improving both medical education and research.

## Data Availability

The dataset that supports the findings of this study is available on request from the corresponding author.
